# Precautions in downstaging for hepatocellular carcinoma with macrovascular invasion before liver transplantation

**DOI:** 10.1097/MS9.0000000000002671

**Published:** 2024-10-22

**Authors:** Li Pang, Lei-bo Xu, Wen-rui Wu

**Affiliations:** a Liver Transplantation Center & Department of Biliary-Pancreatic Surgery, Sun Yat-sen Memorial Hospital, Sun Yat-sen University; b Guangdong Provincial Key Laboratory of Malignant Tumor Epigenetics and Gene Regulation, Sun Yat-sen Memorial Hospital, Sun Yat-sen University, Guangzhou, China

*Dear Editor,*

We reviewed the recently published PLENTY study by Lv *et al*.^1^, which is pioneering in its attempt to utilize neoadjuvant systemic therapy (pembrolizumab plus lenvatinib) in liver transplant (LT) patients with hepatocellular carcinoma (HCC) beyond the Milan criteria, aiming to reduce post-transplant tumor recurrence. The results are encouraging: the 30-month tumor-specific recurrence-free survival (RFS) was 37.5% in the PLENTY group versus 12.5% in the control group, suggesting systemic therapies may be more effective in reducing post-transplant tumor recurrence than locoregional therapies (LRTs). However, the tumor recurrence rate of the PLENTY study is much higher than in other downstaging studies, such as the XXL study (5-year tumor event-free survival of 76.8%)^2^ and UCSF-DS study (5-year post-transplant RFS of 86.1%)^3^. Additionally, nearly half of the patients in the PLENTY study had portal vein tumor thrombus (PVTT), which may contribute to the high recurrence rate. Although macrovascular invasion is no longer an absolute contraindication for LT, careful considerations are needed when downstaging HCC with macrovascular invasion (Fig. 1).

**Figure 1:**
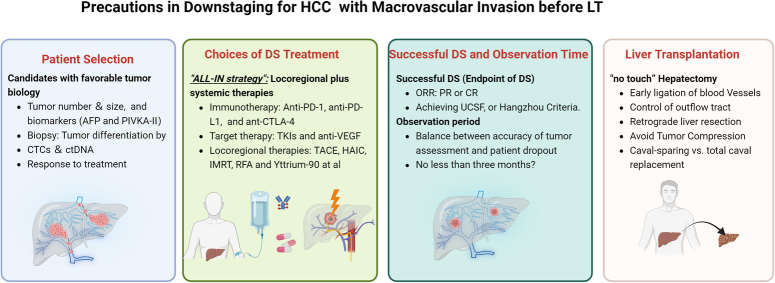
Precautions in downstaging for HCC with macrovascular invasion before LT. HCC, hepatocellular carcinoma; LT, liver transplant.

## Patient selection

Macrovascular invasion in HCC often indicates more aggressive tumor behavior, increasing the likelihood of systemic metastasis. Therefore, stricter selection criteria are necessary for this subset of patients. In addition to parameters such as tumor size, number, and biomarkers (AFP and PIVKA-II), several novel approaches can be considered: (1) tumor biopsy to identify poorly differentiated tumors (referring to extended Toronto criteria); (2) use of circulating tumor cells (CTCs) or DNA (ctDNA) to detect systemic metastasis risks; (3) evaluating response to downstaging (DS) therapies to exclude patients with poor responses, potentially reducing recurrence risk after LT^4^.

## Choices of DS treatment

DS treatment for HCC with macrovascular invasion aims to reduce the extent of the tumor and thrombus to make patients eligible for potentially curative therapies. The PLENTY study suggests that systemic therapy is more effective than LRT in reducing extrahepatic tumor burden, while LRT excels in intrahepatic tumor shrinkage. Therefore, an ‘ALL IN’ downstaging strategy that includes immunotherapy, targeted therapy, and LRT may enhance the success rate of DS and reduce tumor recurrence after LT. Currently, several LT centers are conducting clinical trials employing this strategy^5^. For example, Feng Hao *et al.* are using IMRT combined with PD-1 blockade and lenvatinib for HCC with Vp3 (iPLENTY-pvtt, NCT05339581), and Chao Liu *et al*. are applying LRT in combination with PD-1 blockade and lenvatinib for patients beyond Milan criteria (including Vp1-3). Additionally, Mazzaferro *et al*. reported the preliminary results of ImmunoXXL study at EASL Congress 2024, which involved LT following downstaging with atezolizumab and bevacizumab. After a median follow-up of 8 months, all seven patients were alive with no evidence of disease. These clinical trials will provide valuable evidence for the use of systemic therapies in downstaging HCC before LT.

## Observation period

The timing of transplantation after successful DS is critical. A prolonged wait may lead to tumor recurrence or progression, while too short may not allow adequate assessment of tumor biology. In the PLENTY study, we observed that the median wait time for LT was 33 days in the control group, which was much shorter compared to 114 days in the PLENTY group. Consistently, the 1-year RFS rate of the control group was significantly lower than the PLENTY group (30% vs. 75%). Most DS protocols, including UNOS-DS and UCSF-DS, require a minimum observation period of 90 days, during which no surgery or LRTs are allowed. The relatively short observation period in the PLENTY study, particularly in the control group, may have contributed to the higher recurrence rate. A comprehensive evaluation of treatment efficacy and tumor biology is essential for HCC patients with macrovascular invasion.

## ‘No touch’ hepatectomy

The ‘no touch’ technique aims to minimize the risk of tumor seeding and dissemination during hepatectomy by avoiding direct manipulation of the tumor and its surrounding tissues. The ‘no touch’ procedure typically begins with the dissection of the liver, followed by clamping the inflow vessels (hepatic artery and portal vein) and outflow vessels (infrahepatic and suprahepatic inferior vena cava). After securing these vessels, the liver is further dissected and removed. Zheng *et al*. found that the ‘no touch’ technique improved prognosis in HCC patients with PVTT compared to conventional methods^6^. Additionally, Pravisani *et al*.^7^ reported worse outcomes with caval-sparing recipient hepatectomy compared to total caval replacement in LT for HCC.

## Ethical approval

Not applicable.

## Consent

Not applicable.

## Source of funding

National Natural Science Foundation of China, No. 82203747; China Postdoctoral Science Foundation, Nos. 2022TQ0388 and 2023M734036.

## Author contribution

L.P. and W.W.: contributed to the idea, writing, and proofreading; L.X.: provided supervision and approval.

## Conflicts of interest disclosure

The authors declare no conflicts of interest.

## Research registration unique identifying number (UIN)

Not applicable.

## Guarantor

Wen-rui Wu, MD, PhD, Associated Professor; Liver Transplantation Center & Department of Biliary-Pancreatic Surgery, Sun Yat-sen Memorial Hospital, Sun Yat-sen University, 510120 Guangzhou, China; Address: No. 107, Yan Jiang Xi Road, Guangzhou, China; E-mail: wuwr6@mail.sysu.edu.cn

## Data availability statement

There were no new data generated.

## Provenance and peer review

Not available.
